# DNA metabarcoding reveals the seasonal variation of dietary composition of Taihangshan macaque (*Macaca mulatta tcheliensis*), Jiyuan, north China

**DOI:** 10.1002/ece3.11256

**Published:** 2024-04-19

**Authors:** Yanyan Zhou, Chunbo Liu, Jundong Tian, Qi Shao, Jiqi Lu

**Affiliations:** ^1^ School of Life Sciences Zhengzhou University Zhengzhou China; ^2^ Institute of Biodiversity and Ecology Zhengzhou University Zhengzhou China

**Keywords:** dietary composition, dietary diversity, DNA metabarcoding, seasonal variation, Taihangshan macaque (*Macaca mulatta tcheliensis*)

## Abstract

Dietary analysis in wildlife is fundamental for understanding their flexible response to seasonal changes and developing effective conservation management measures. Taihangshan macaque (*Macaca mulatta tcheliensis*) is the northernmost population of rhesus macaque, currently only distributed in the southern Mt. Taihangshan area. This area belongs to a semi‐arid region resulting in limited plant food availability for Taihangshan macaques, with seasonal variation. Herein, we used a chloroplast *trnL* DNA metabarcoding approach to identify the plant diet diversity and composition from 100 fecal samples of Taihangshan macaque in four seasons (spring, summer, autumn, and winter) from 2020 to 2021. The results revealed that (1) a total of 48 distinct families, 88 genera, and 52 species within the 105 food items that were consumed by Taihangshan macaques throughout the year; (2) the diversity of food items exhibited significant differences across the four seasons; (3) Rosaceae, Rhamnaceae, Fagaceae, and Poaceae are the preferential food items for Taihangshan macaques and have different relative abundances, fluctuating with seasonal variation. DNA metabarcoding can expand our understanding of the repertoire of food items consumed by Taihangshan macaques by detecting some consumed food items in this population that were not yet discovered using traditional methods. Therefore, the integrative results from traditional methods and DNA metabarcoding can provide a fundamental understanding of dietary composition to guide the conservation management of Taihangshan macaques.

## Graphical abstract

Based on DNA metabarcoding, we identified 105 plant items of 48 families from fecal samples of Taihangshan macaque across spring, summer, autumn, and winter seasons from 2020 to 2021. The dominant families included Rosaceae, Poaceae, Rhamnaceae, and Fagaceae. The dietary composition of Taihangshan macaques was shifted by the seasonal changes and food availability.

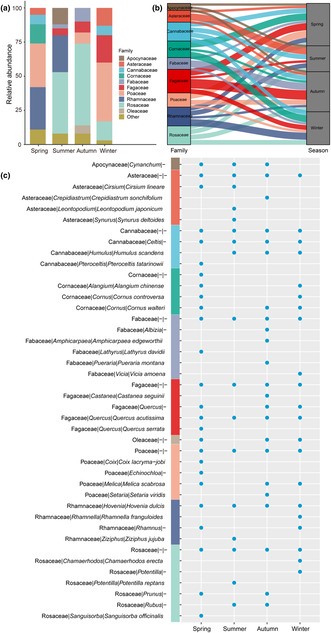

## 1 INTRODUCTION

Food availability undoubtedly affects the survival and health of wild animals, especially mammals under extreme environmental conditions (Guo et al., 2021; Trevelline & Kohl, 2022). Dietary analysis in wildlife is fundamental for understanding the flexible response of animals to seasonal changes (Quemere et al., 2013) and developing effective conservation management measures (Kraus et al., 2019; Martin, 1987; Shrestha et al., 2018). Traditionally employed methods, such as direct observation in the field and microscopic examination from fecal samples or stomach contents (Hill, 1997; Zhang et al., 2022), suffer from weak taxonomic resolution and/or limitations due to species feeding habitats that are difficult to observe. Notably, DNA metabarcoding provides a comprehensive analysis of dietary composition and variation (Browett et al., 2023; Cabodevilla et al., 2021; Kartzinel & Pringle, 2020; Lazic et al., 2021; Reese et al., 2019), and it enables us to accurately detect dietary profiles at population scale or at the individual level (Kennedy et al., 2020; Mishra et al., 2016; Taberlet et al., 2007; Tang et al., 2021).

Rhesus macaque (*Macaca mulatta*) is the most widely distributed non‐human primate species in the world (Fooden, 1982, 2000) and occupies diverse habitats in China from tropical forests to temperate snow mountains (Jiang et al., 1991; Lu, 2020). As an opportunistic and generalist primate species, rhesus macaques are omnivorous and feed mainly on plants, occasionally insects, and small vertebrates (Lu et al., 2007; Lyv et al., 2002). Taihangshan macaque (*M. m. tcheliensis*) is one of the subspecies of Chinese rhesus macaque, and is the northernmost population and endemic to China, currently restricted to the southern Mt. Taihangshan area, temperate China (Lu, 2020). Compared with other subspecies of Chinese rhesus macaque, Taihangshan macaque has unique genetic variation (Liu et al., 2018), lower genetic diversity (Zhou et al., 2023), and genetic vulnerability in the future (Wu et al., 2023).

Mt. Taihangshan locates at the eastern edge of the Chinese Loess Plateau and north of the Yellow River, belongs to a semi‐arid region, and is influenced by the continental monsoon climate (Lu, 2020). The vegetation in Mt. Taihangshan is dominated by natural secondary forests, and its growth and development stages are greatly affected by temperature and sunshine (Wang et al., 2020). Furthermore, the plant resources availability (such as the quantity and quality) is limited for Taihangshan macaques with the seasonal variation, which can threaten their survival, especially during winter and early spring when food is sparse and the temperatures drop significantly (Lu, 2020; Lu et al., 2007; Lyv et al., 2002).

Previous studies of dietary composition on Taihangshan macaques have been conducted using methods such as direct observation (Cui et al., 2019; Lyv et al., 2002; Shao et al., 2023) and microscopic examination (Guo et al., 2011). Here, the results are mainly focused on the food types (seeds, leaves, roots, flowers, fruits, and young bark, etc.), corresponding to the seasonal variation, with varying nutrient requirements (Cui et al., 2019, 2020; Shao et al., 2023). Moreover, it is possible that some food items consumed by Taihangshan macaques have not yet been identified using these methods. Therefore, it is necessary to conduct a detailed investigation into the variation of plant dietary diversity and the feeding preference of Taihangshan macaques, which could provide a valuable insight for protecting this wild population. Herein, we collected the fecal samples of Taihangshan macaque in four seasons and used DNA metabarcoding to (1) estimate the diversity of food items of Taihangshan macaques and (2) identify the dietary composition and preference of this population.

## 2 MATERIALS AND METHODS

### 2.1 Study area and sample collection

**FIGURE 1:**
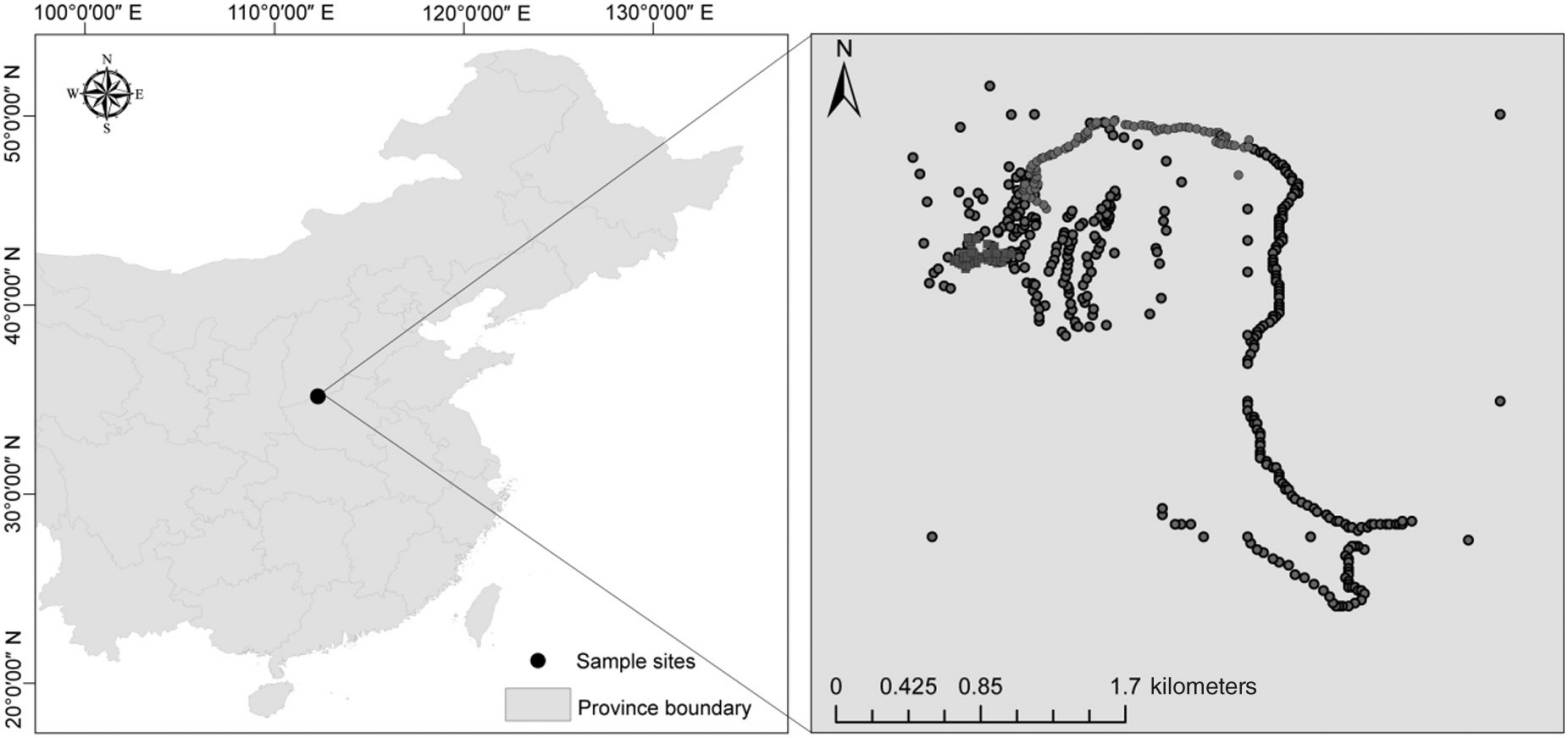
Sampling site of the Taihangshan macaques. The left map shows the location of this study in China. The right map was the home range of the Wangwu troop‐1 of Taihangshan macaques in the Mt. Taihangshan area. The dots represented where Taihangshan macaques were observed in the field throughout the year. The fecal samples of Taihangshan macaques were collected from their home range area for dietary analysis.

Fieldwork was conducted on the Taihangshan Macaque National Nature Reserve (TMNNR) (34°54′–35°42′ N, 112°02′–113°45′ E) (Figure 1), a protected area located in the southern Mt. Taihangshan. This area has four distinctive seasons (Lu, 2020; Lu et al., 2007) including spring (from March to May), summer (from June to August), autumn (from September to November), and winter (from December to February of the next year) (Lu, 2020; Shao et al., 2023). The fecal samples were collected from a free‐ranging troop of Taihangshan macaque (Wangwu‐1), which spanned the four seasons from 2020 to 2021. The home range of this group is approximately 24 km^2^ (Cui et al., 2019), and they have been identified since 2005 and consist of 100 individuals, including seven adult males, 28 adult females, 43 juveniles, and 22 infants (Shao et al., 2023). However, only 157 fecal samples of Taihangshan macaque were collected due to macaque individuals were hard to be traced and extremely sensitive to human presence in the field. The fecal samples were transported in a cooled icebox at 4°C to the laboratory and then stored at −20°C until DNA extraction.

### 2.2 DNA extraction and sequencing

Total DNA was extracted from fecal samples of Taihangshan macaque using the TIANamp Stool DNA Kit (TIAGEN, China). The quality of DNA was quantified using the NanoDrop 2000 Spectrophotometer (Thermo Fisher Scientific, Wilmington, DE, USA). The pair primers c (5′‐CGAAATCGGTAGACGCTACG‐3′) and h (5′‐CCATTGAGTCTCTGCACCTATC‐3′) were used to amplify the partial sequences of chloroplast *trnL* (150 bp), which was considered a suitable molecular marker for plant species identification (Taberlet et al., 2007). Samples were individually indexed using a barcode for the *trnL* amplicons of each sample. Three independent polymerase chain reactions (PCR) were performed for each sample. The 25 μL total volume of PCR mixture consisted of 3 μL DNA template, 3 μL of both forward and reverse primers (1.5 μL each), and 19 μL of T3 Super PCR mix (Tsingke). The amplification reaction was performed under the following program: 94°C for 3 min, followed by 30 cycles of denaturation at 94°C for 30 s, annealing at 56°C for 45 s, extension at 72°C for 45 s, and a final extension step at 72°C for 10 min. In parallel, a blank PCR of each primer pair was included as a negative control to monitor the laboratory contamination. Out of a total of 157 fecal samples, we were able to amplify 100 samples (25 for each season) due to the low quality of fecal samples. The resulting PCR amplicons were purified using the TIANamp Universal DNA Purification Kit (TIAGEN, China) and quantified using a NanoDrop 2000 Spectrophotometer (Thermo Fisher Scientific, Wilmington, DE, USA). The indexed amplicons were then pooled into four libraries according to four seasons and sequenced by Novogene Co., Ltd. (Beijing, China) on an Illumina NovaSeq 6000 platform (2 × 150 bp paired‐end reads).

### 2.3 Constructing the local reference database

To identify taxonomic information of plant sequences from fecal samples, we established a DNA reference database from the plant species recording in the book of Taihangshan Macaques Nature Reserve Scientific Investigation Collection (Song & Qu, 1996). We queried the NCBI database for records of plant‐matching “*trnL*” sequences at the species level. A total of 146 families of 1458 plant species sequences matching these search terms were obtained from the NCBI database in October 2021.

### 2.4 Sequence processing and bioinformatics

The sequence demultiplexing and preliminary identifications were conducted by QIIME v1.9.1 (Caporaso et al., 2010). Demultiplexing used split_libraries_fastq.py (Kuczynski et al., 2011). Paired‐end reads were merged using VSEARCH v2.8 (Rognes et al., 2016) with parameter “‐‐fastq_mergepairs”, and then, all merged sequences of each sample were pooled. Adaptor and primer regions were removed using VSEARCH v2.8 with “‐‐fastx_stripleft, ‐‐fastx_stripright.” All sequences shorter than 100 bp were removed using USEARCH v11 (Edgar, 2013). Quality‐filtered sequences were then deduplicated using VSEARCH v2.8 with “‐‐derep_fulllength 2.” Potential chimeras and singletons (with an abundance of less than 20 reads) were removed using VSEARCH v2.8. The retained sequences were clustered into operational taxonomic units (OTUs) at 97% similarity using VSEARCH v2.8 with “‐‐cluster_fast, ‐‐id 0.97.” The representative sequences from each OTU were annotated using BLASTN v2.7.1 (Camacho et al., 2009) against the reference taxonomy database under the algorithm of the lowest common ancestor (LCA). OTUs were refined to the taxonomic level with the following percentage homology thresholds: species‐, genus‐, and family‐level identifications were assigned at a minimum of ≥99%, 98% to <99%, and 97% to <98% similarity, respectively. Moreover, when one sequence was associated with more than one taxon, it was assigned to the higher taxonomic level (genus or family).

To gain an integrative understanding of plant species richness, diversity, and dominance throughout the year, alpha diversity matrices (i.e., Chao1, Shannon, and Simpson) were performed based on the OTU level using Mothur (Schloss et al., 2009) and displayed using R v.3.6 (R Core Team, 2018). To evaluate the pattern of dispersion of samples within each season, beta diversity was calculated via Euclidean distance (Tang et al., 2021) using USEARCH v11 and visualized by principal component analysis (PCA). Diversity was compared between different seasons to assess temporal differences in diet composition throughout the year. R was used to visualize the variations in relative abundance and composition of diet taxa sequence across seasons as a histogram and Sankey plot.

## 3 RESULTS

### 3.1 Taxonomic assignment and diversity of diet

**FIGURE 2:**
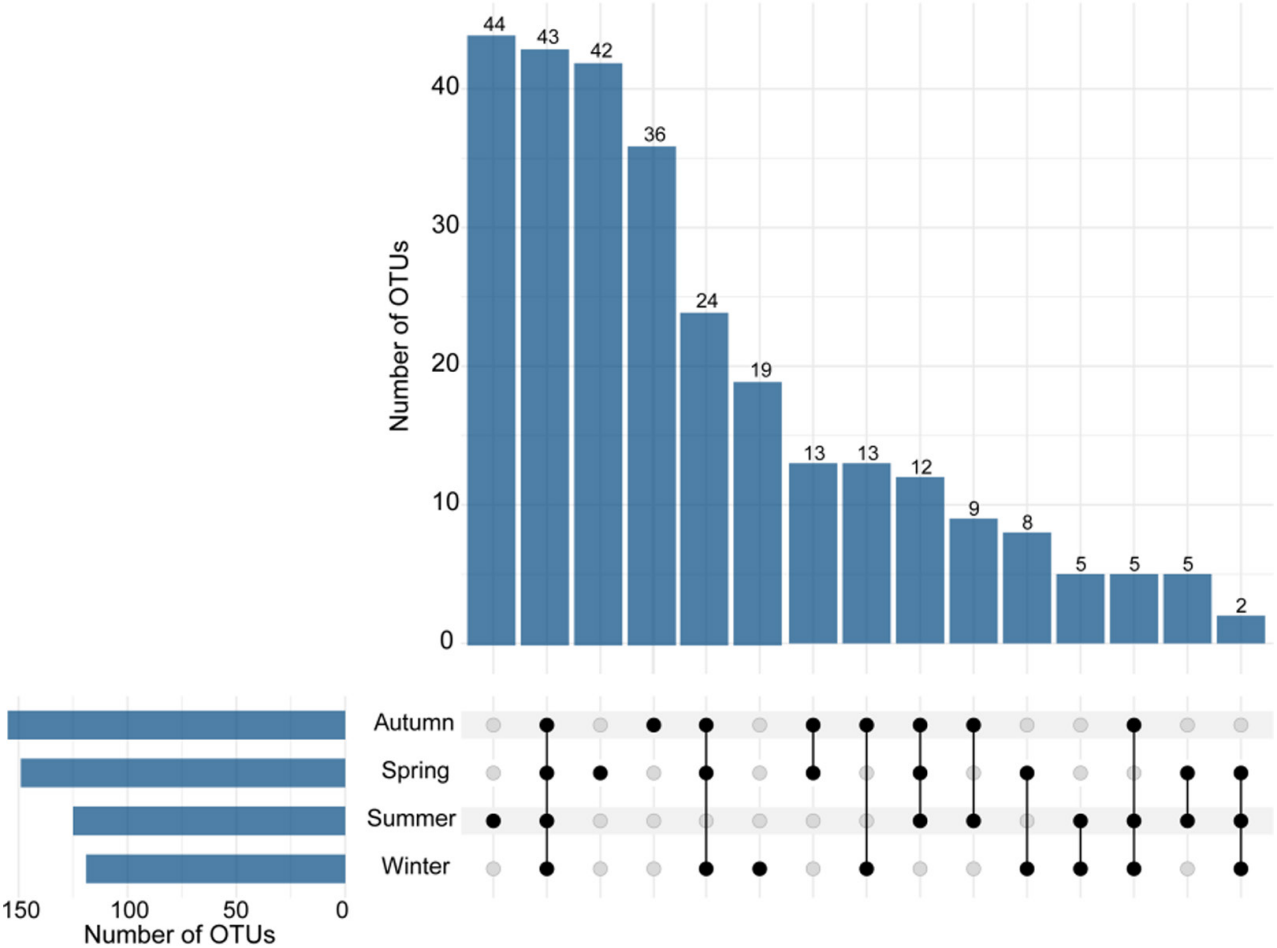
Distribution of operational taxonomic units (OTUs) in each season. The bar chart at the bottom left represents the number of OTUs in different seasons. The dotted line at the bottom right shows the intersection within different seasons. The bar chart above represents the number of OTUs in each type of intersection within different seasons.

The diet data included a total of 2,119,522 high‐quality sequences (310,002–703,505) and 79,763 unique sequences after removal of singletons (18,434–23,756) in four seasons (Table 1). This showed that the number of OTUs was the highest in autumn, followed by spring and summer, and winter has the lowest one (Figure S1). For instance, the number of OTUs was 149 for spring, 125 for summer, 155 for autumn, and significantly decreased to 119 in winter (Table 1). The shared OTUs in four seasons was 15.36% (43), three seasons was 15.36% (43), two seasons was 18.93% (53), and 50.35% (141) of the OTUs were present in only one season (Figure 2). In total, we identified 48 potential families, 88 genera, and 52 species within the 105 food items that were consumed by Taihangshan macaques in the four seasons (Table S1). Peak diversity in the consumption of food items at the family level was detected in spring, followed by autumn, summer, and then winter (Table 1).

**TABLE 1:** Number of sequences, operational taxonomic units (OTUs), and identified food items in Taihangshan macaques diet throughout the year.

Seasons	Sequences	Unique sequences after removal of singleton	OTUs	Family	Food items
Spring	703,505	23,756	149	34	56
Summer	572,229	18,434	125	25	41
Autumn	310,002	18,979	155	28	51
Winter	533,786	18,594	119	23	37

**FIGURE 3:**
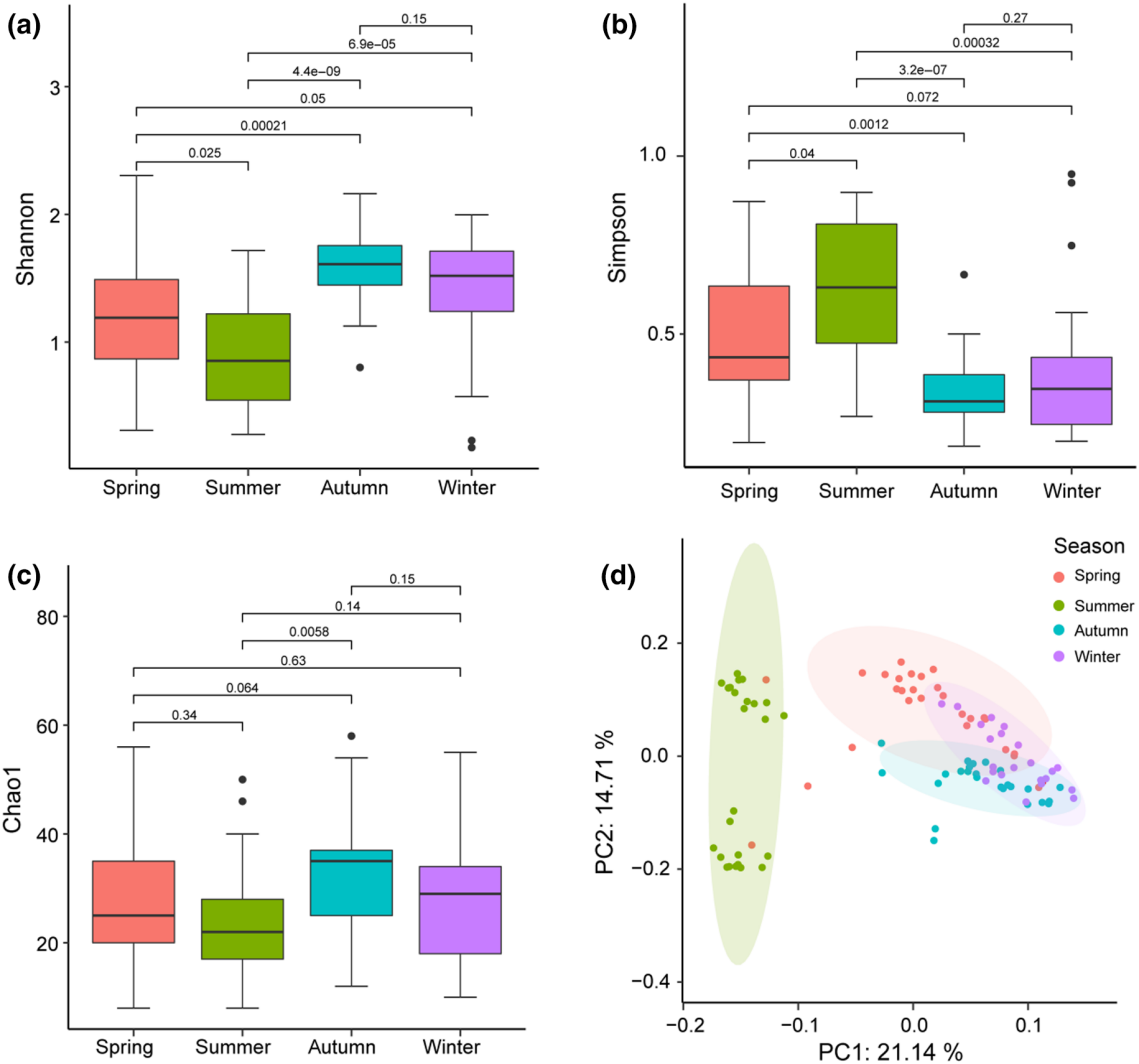
Dietary diversity and clustering patterns of Taihangshan macaques in four seasons. Box‐and‐whisker plots for alpha diversity in plant food items estimators (a) Shannon, (b) Simpson, and (c) Chao1 indices. Different values are labeled between seasons (*t*‐test); and (d) Seasonal clustering patterns in diet composition of Taihangshan macaques. The differences in the dietary composition between seasons have been determined by beta diversity, which are colored by season. Ellipses show 95% of data rotated to the direction of maximum spread.

Alpha diversity indices (Shannon, Simpson, and Chao1) indicated seasonal differences in the diversity of food items of Taihangshan macaque (Figure 3). There were significant differences in the Shannon and Simpson Diversity Index values among the four seasons (*p* < .05), except between winter and autumn/spring (Figure 3a,b). However, no significant differences were found in the Chao1 index of food items among the four seasons, except for between summer and autumn (Figure 3c). Moreover, the two‐dimensional PCA provides additional corroborative evidence for seasonal differences in the dietary composition of Taihangshan macaques (Figure 3d). In the PCA, the *x*‐axis separated the summer and the other three seasons, which explained 21.14% of the total variation. The *y*‐axis identified a high degree of inter‐season variability within summer food items, which explains 14.71% of the total variance.

### 3.2 Dietary composition and variation across seasons

**FIGURE 4:**
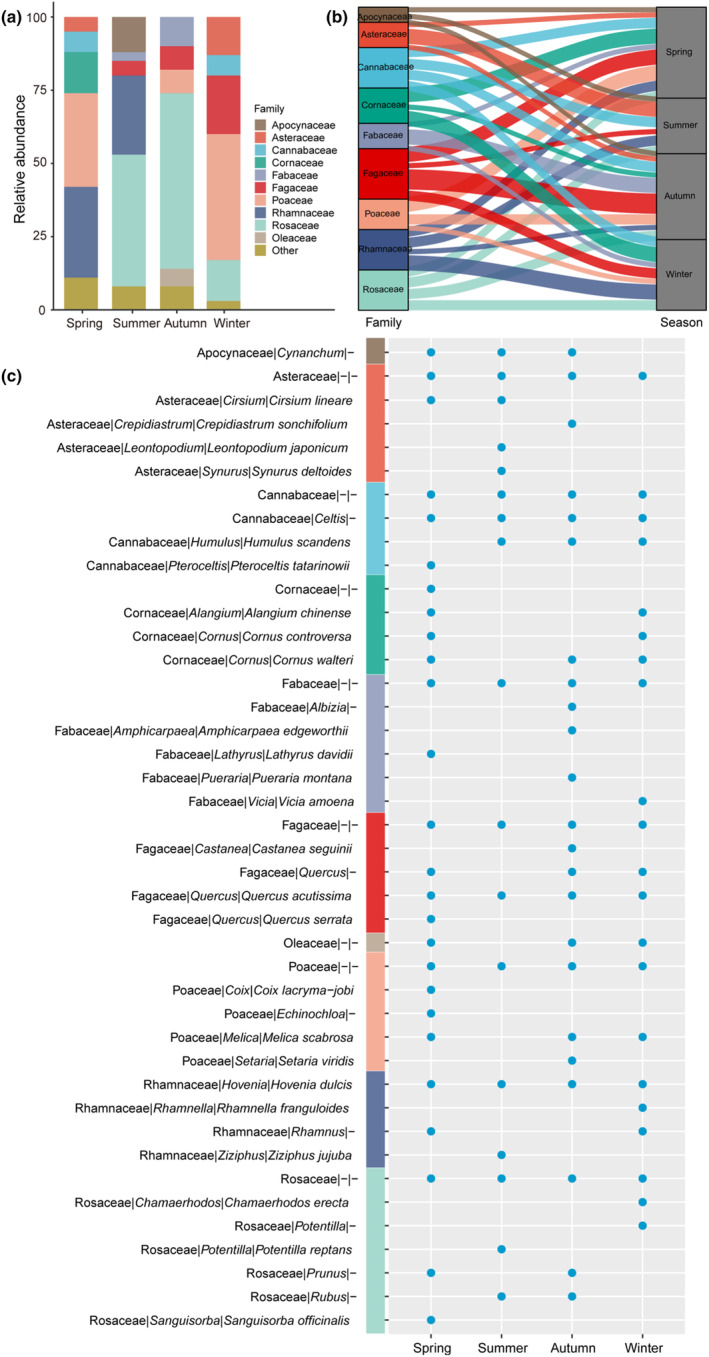
Seasonal changes in the dietary composition of Taihangshan macaques. (a) Barplot showing the top 10 relative abundance of family‐level taxa in spring, summer, autumn, and winter. Low abundance taxa (outside of the top 10 families) were grouped together as “Others”; (b) Indicator families that were related to each season and tracked using Sankey plots. Line width was scaled to reflect the number of genera within each family (higher indicator value of families represented by more genera in a season), which were colored by family; and (c) the taxonomic identification of family|genus|species of the top 10 family‐level food items in each season. “‐” represented the absent taxa information.

Many different plant food items were consumed by Taihangshan macaques and great seasonal variation was exhibited (Figure 4; Table S1). The highest relative abundance was Poaceae in spring and winter, and Rhamnaceae was also high in spring and summer (Figure 4a). Furthermore, we found a trend in the consumption of Rosaceae that shifted from a higher number during summer and autumn to lower levels during winter, to the lowest in spring. Moreover, Fagaceae was consumed by Taihangshan macaques and gradually increased from spring to winter (Figure 4a,b). Meanwhile, we also found 3%–11% of food items at the family level with lower frequencies and abundances, which were grouped into the “Others” category (Figure 4a). Additionally, some food items within dominant families (such as Rosaceae, Rhamnaceae, Fagaceae, and Cannabaceae) were identified at the genus level in four seasons (Figure 4b). The top 10 families consisted of 48 food items, and exhibited a lower resolution of taxonomic information, classifiable at the family (27.08%), genus (20.83%), and species level (52.09%) (Figure 4c). Similarly, the low abundance taxa (Others) is composed of 57 food items that were identified and revealed at the family (29.82%), genus (22.81%), and species level (47.37%) (Figure S2).

## 4 DISCUSSION

By using DNA metabarcoding data from chloroplast *trnL* from 100 fecal samples of Taihangshan macaque, we provide a detailed scenario of the plant dietary diversity and composition of this population in four seasons (spring, summer, autumn, and winter). We identified a total of 48 potential families, 88 genera, and 52 species within the 105 food items that were consumed by Taihangshan macaques throughout the year. The diversity of food items of Taihangshan macaques exhibited significant differences between the four seasons, and there was a high degree of inter‐season variation within summer. Furthermore, Rosaceae, Rhamnaceae, Fagaceae, and Poaceae are the preferred food items for Taihangshan macaques and have different relative abundance fluctuations, with seasonal variation.

A significant difference was detected in the number and alpha diversity of plant food items in the four seasons, demonstrating seasonal shifts in the food availability of Taihangshan macaques. Food availability is one of the most crucial factors affecting the survival of wildlife in harsh climate conditions (Goldberg et al., 2020; Huang et al., 2021). Investigations into the wild forest resources have revealed that arbor, shrub, and lianas are the dominant plant taxa in southern Mt. Taihangshan, with shrub, especially identified as the most abundant (Lu & Chen, 2020; Zhang et al., 2023). Moreover, the growth stages, diversity, and relative abundances of these plant taxa were affected by temperature, sunshine, and humidity in the different seasons (Chen, 2021; Huang et al., 2020; Shao et al., 2023; Wang et al., 2021). Additionally, the plant species in southern Mt. Taihangshan supported the “mid‐domain effect” model, which has the highest abundance value of plant species at the altitude is between 900 and 1100 m (Lu & Chen, 2020). Interestingly, Taihangshan macaques with a strong migration ability has the highest frequency of food‐seeking activities on the hillside of altitudes 500–1400 m (Lu et al., 2007; Lyv et al., 2002). Therefore, we are reasonable to deduce that the variation in the dietary composition potentially arises from the availability of food resources with seasonal variation in the home ranges of Taihangshan macaques.

Some plants were consumed continuously (different parts may be eaten throughout the year), while the consumption of others was associated with particular seasons. For instance, Rosaceae, Rhamnaceae, Fagaceae, and Poaceae are the preferred food items for Taihangshan macaques and have different relative abundance fluctuated with seasonal variation. Rosaceae, Rhamnaceae, and Fagaceae mainly belong to arbor and shrub, which can provide leaves, fruits, and seeds for Taihangshan macaques during different growth stages (Cui et al., 2019; Shao et al., 2023). Rosaceae has a high abundance in southern Mt. Taihangshan areas (Lu & Chen, 2020) and is a dominant food source for Taihangshan macaques. Therefore, the plant taxa within the Rosaceae family can provide an abundance of seeds and fruits including total energy intake, carbohydrate, fat, and available protein for Taihangshan macaques in different seasons (Cui et al., 2019). Likewise, Poaceae was available in winter and spring, but low in autumn, which confirmed that plant growth stages were the main driving factor for the variation of dietary composition and accordance with the optimal foraging theory (Pyke et al., 1977). Meanwhile, other dominant families, such as Rhamnaceae, Fagaceae, and Poaceae, can also provide different types of food items to Taihangshan macaques corresponding to seasonal variation. Moreover, dietary composition evidence suggests that Taihangshan macaque can regulate the macronutrient intake by consuming other plant food items making up for the energy deficiency (Cui et al., 2019). For example, in the case of snow cover and frozen soils in winter and early spring, the drop in available food resources could make it challenging for Taihangshan macaques to obtain sufficient nutrients (Lu, 2020; Lu et al., 2007). Consequently, some foods were available to be eaten only at low levels and in a single season (such as Asteraceae, Cannabaceae, and Others), which had shifted Taihangshan macaques diet to some other families for meeting high‐energy requirements at low temperatures (Cui et al., 2019; Tang et al., 2021). This deduction has initially been articulated based on wild observation methods (Guo et al., 2011; Lu et al., 2007; Lyv et al., 2002) and was later confirmed via macronutrient investigation (Cui et al., 2018, 2019). It is worth noting that the food resource availability has a high degree of inter‐season variability within summer. Leaves, fruits, and flowers are the main food items for Taihangshan macaques in summer (Cui et al., 2019; Shao et al., 2023). This diet can be provided by the dominant families (Rosaceae and Rhamnaceae), which might limit the availability of other plant food items intake in summer (Lyv et al., 2002). Alternatively, this was likely caused by the imperfect sampling strategy due to this species always hiding in dense forests and valleys during the summer period. These demonstrated that Taihangshan macaques had the capacity to shift their dietary preferences according to food availability.

In investigating the plant diet of Taihangshan macaques, DNA metabarcoding assay detected many food items at the family/genus/species level (such as Amaryllidaceae, Apocynaceae, and Vitaceae) that had not been observed or identified in previous studies (Guo et al., 2011; Lu et al., 2007; Lyv et al., 2002; Shao et al., 2023). Cannabaceae was an important dietary component for Taihangshan macaques, especially in spring and winter but was not detected by microscopic examination (Guo et al., 2011), which is possibly due to plant being highly digested or a small sample size that was not easily identified using microscopy. However, Ranunculaceae and Papaveraceae at relatively low frequencies in diet of Taihangshan macaques being observed from direct observation (Shao et al., 2023) were not found in DNA metabarcoding. One possible explanation is that our reference database has some limitations for the investigation of the diet composition of Taihangshan macaque, which missed some taxonomic information in this study. The second one is that, Taihangshan macaques may employ a flexible foraging strategy, in which they could shift the dietary composition corresponding to the relative availability of food items in different years.

In summary, the dietary composition and preference of Taihangshan macaques were influenced by the food availability in different seasons. Although DNA metabarcoding has a low resolution with one marker (*trnL*), it also expands the identified repertoire of food items consumed by Taihangshan macaques. Therefore, the integrative results from traditional methods and DNA metabarcoding can provide the fundamental data of conservation management for Taihangshan macaques.

## AUTHOR CONTRIBUTIONS

**Yanyan Zhou:** Conceptualization (equal); formal analysis (equal); methodology (equal); software (equal); writing – original draft (equal); writing – review and editing (equal). **Chunbo Liu:** Conceptualization (equal); formal analysis (equal); methodology (equal); software (equal); writing – original draft (equal). **Jundong Tian:** Methodology (supporting); resources (supporting). **Qi Shao:** Investigation (supporting); methodology (supporting); resources (supporting). **Jiqi Lu:** Conceptualization (equal); funding acquisition (equal); resources (equal); supervision (equal); writing – review and editing (equal).

## CONFLICT OF INTEREST STATEMENT

The authors declare that they have no conflict of interest.

## Supporting information


Data S1

## Data Availability

Data sequences in this study were deposited into the NCBI Sequence Read Archive (SRA) under accession number: PRJNA1006695.
