# Supplementary material for: DNA metabarcoding reveals the seasonal variation of dietary composition of Taihangshan macaque (Macaca mulatta tcheliensis), Jiyuan, north China
Source: Ecol Evol. 2024 Apr 19;14(4):e11256. doi: 10.1002/ece3.11256 (PMC11027012; doi:10.1002/ece3.11256)
Supplement: Supplementary file 1 — Data S1 [file ECE3-14-e11256-s001.pdf]

Table S1. Plant diet composition of Taihangshan macaque in the Mt. Taihangshan area from September 2020 to August 2021

Family	Food items	Level of identification	Spring	Summer	Autumn	Winter
Adoxaceae	*Viburnum sp.*	Genus	✓		✓	
Adoxaceae	*Viburnum schensianum*	Species	✓		✓	
Amaranthaceae	*Chenopodium giganteum*	Species		✓	✓	
Amaryllidaceae	*Allium sp.*	Genus	✓	✓	✓	✓
Anacardiaceae	*Anacardiaceae sp.1*	Family	✓	✓		
Anacardiaceae	*Toxicodendron sp.1*	Genus				✓
Anacardiaceae	*Toxicodendron sp.2*	Genus				✓
Anacardiaceae	*Anacardiaceae sp.2*	Family			✓	
Anacardiaceae	*Pistacia chinensis*	Species			✓	
Anacardiaceae	*Toxicodendron succedaneum*	Species			✓	
Apocynaceae	*Cynanchum sp.*	Genus	✓	✓	✓	
Araliaceae	*Hedera nepalensis*	Species	✓			
Asparagaceae	*Asparagaceae sp.*	Family	✓	✓		
Asteraceae	*Asteraceae sp.1*	Family	✓	✓		
Asteraceae	*Cirsium lineare*	Species	✓	✓		
Asteraceae	*Asteraceae sp.2*	Family			✓	✓
Asteraceae	*Crepidiastrum sonchifolium*	Species			✓	
Asteraceae	*Synurus deltoides*	Species		✓		
Asteraceae	*Leontopodium japonicum*	Species		✓		
Betulaceae	*Betulaceae sp.*	Family	✓			
Bignoniaceae	*Catalpa ovata*	Species			✓	
Cannabaceae	*Celtis sp.*	Genus	✓	✓	✓	✓

Cannabaceae	*Cannabaceae sp.*	Family	✓	✓	✓	✓
Cannabaceae	*Pteroceltis tatarinowii*	Species	✓			
Cannabaceae	*Humulus scandens*	Species		✓	✓	✓
Caprifoliaceae	*Lonicera sp.*	Genus	✓	✓	✓	✓
Caryophyllaceae	*Stellaria media*	Species				✓
Celastraceae	*Celastraceae sp.*	Family	✓			✓
Celastraceae	*Euonymus sp.*	Genus			✓	
Celastraceae	*Celastrus rosthornianus*	Species		✓	✓	
Commelinaceae	*Commelina benghalensis*	Species			✓	
Commelinaceae	*Streptolirion volubile*	Species			✓	
Convolvulaceae	*Convolvulus arvensis*	Species		✓		
Cornaceae	*Cornus walteri*	Species	✓		✓	✓
Cornaceae	*Cornus controversa*	Species	✓			✓
Cornaceae	*Alangium chinense*	Species	✓			✓
Cornaceae	*Cornaceae sp.*	Family	✓			
Crassulaceae	*Hylotelephium sp.*	Genus	✓	✓		
Cupressaceae	*Cupressaceae sp.*	Family	✓		✓	
Dioscoreaceae	*Dioscorea sp.*	Genus	✓			
Ericaceae	*Rhododendron concinnum*	Species				✓
Fabaceae	*Fabaceae sp.1*	Family	✓	✓		
Fabaceae	*Lathyrus davidii*	Species	✓			
Fabaceae	*Fabaceae sp.2*	Family			✓	✓
Fabaceae	*Vicia amoena*	Species				✓
Fabaceae	*Albizia sp.*	Genus			✓	
Fabaceae	*Pueraria montana*	Species			✓	
Fabaceae	*Amphicarpaea edgeworthii*	Species			✓	

Fagaceae	*Quercus acutissima*	Species	✓	✓	✓	✓
Fagaceae	*Fagaceae sp.*	Family	✓	✓	✓	✓
Fagaceae	*Quercus sp.1*	Genus	✓		✓	✓
Fagaceae	*Quercus serrata*	Species	✓			
Fagaceae	*Quercus sp.2*	Genus			✓	
Fagaceae	*Castanea seguinii*	Species			✓	
Grossulariaceae	*Ribes fasciculatum*	Species	✓			
Hamamelidaceae	*Sinowilsonia henryi*	Species	✓			
Juglandaceae	*Juglandaceae sp.*	Family	✓			
Juglandaceae	*Juglans mandshurica*	Species		✓		
Lamiaceae	*Lamiaceae sp.*	Family	✓			
Lardizabalaceae	*Akebia sp.*	Genus			✓	
Lardizabalaceae	*Akebia trifoliata*	Species			✓	
Lythraceae	*Lythraceae sp.*	Family			✓	
Malvaceae	*Grewia biloba*	Species		✓	✓	✓
Moraceae	*Broussonetia papyrifera*	Species	✓	✓		✓
Moraceae	*Morus sp.*	Genus		✓		
Moraceae	*Moraceae sp.*	Family		✓		
Oleaceae	*Oleaceae sp.*	Family	✓		✓	✓
Onagraceae	*Oenothera sp.*	Genus				✓
Orchidaceae	*Orchidaceae sp.*	Family	✓			
Oxalidaceae	*Oxalis corniculata*	Species		✓		
Pinaceae	*Pinaceae sp.*	Family	✓		✓	
Poaceae	*Melica scabrosa*	Species	✓		✓	✓
Poaceae	*Poaceae sp.3*	Family	✓			✓
Poaceae	*Poaceae sp.1*	Family	✓	✓		

Poaceae	*Echinochloa sp.*	Genus	✓			
Poaceae	*Coix lacryma-jobi*	Species	✓			
Poaceae	*Poaceae sp.2*	Family		✓	✓	✓
Poaceae	*Setaria viridis*	Species			✓	
Polygonaceae	*Polygonaceae sp.*	Family	✓	✓		✓
Polygonaceae	*Polygonum sp.*	Genus	✓	✓	✓	
Polygonaceae	*Fallopia sp.*	Genus	✓	✓	✓	
Polygonaceae	*Polygonum viviparum*	Species	✓			
Primulaceae	*Lysimachia hemsleyana*	Species		✓		
Rhamnaceae	*Hovenia dulcis*	Species	✓	✓	✓	✓
Rhamnaceae	*Rhamnus sp.*	Genus	✓			✓
Rhamnaceae	*Rhamnella franguloides*	Species				✓
Rhamnaceae	*Ziziphus jujuba*	Species		✓		
Rosaceae	*Prunus sp.*	Genus	✓		✓	
Rosaceae	*Rosaceae sp.1*	Family	✓	✓		
Rosaceae	*Sanguisorba officinalis*	Species	✓			
Rosaceae	*Chamaerhodos erecta*	Species				✓
Rosaceae	*Rosaceae sp.2*	Family			✓	✓
Rosaceae	*Potentilla sp.*	Genus				✓
Rosaceae	*Potentilla reptans*	Species		✓		
Rosaceae	*Rubus sp.*	Genus		✓	✓	
Rubiaceae	*Paederia cavaleriei*	Species		✓		
Rubiaceae	*Emmenopterys henryi*	Species		✓		
Rutaceae	*Citrus trifoliata*	Species	✓			
Sapindaceae	*Aesculus chinensis*	Species	✓	✓	✓	✓
Sapindaceae	*Koelreuteria paniculata*	Species	✓		✓	

Simaroubaceae	*Ailanthus altissima*	Species			✓	
Solanaceae	*Solanaceae sp.*	Family	✓	✓		✓
Ulmaceae	*Ulmaceae sp.1*	Family	✓		✓	
Ulmaceae	*Ulmaceae sp.2*	Family			✓	✓
Vitaceae	*Vitaceae sp.*	Family	✓	✓	✓	✓

Note: The number (1,2,3) in the table represent different plant species under the same family (genera)

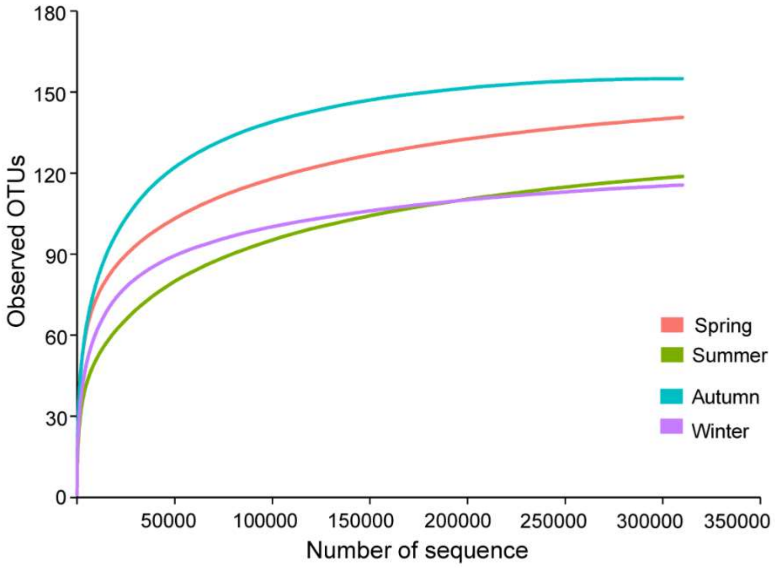

Figure S1 The rarefaction curve of the *trnL* gene sequence for different seasons of plant food items of Taihangshan macaques calculated for operational taxonomic units (OTUs) at 97% similarity.

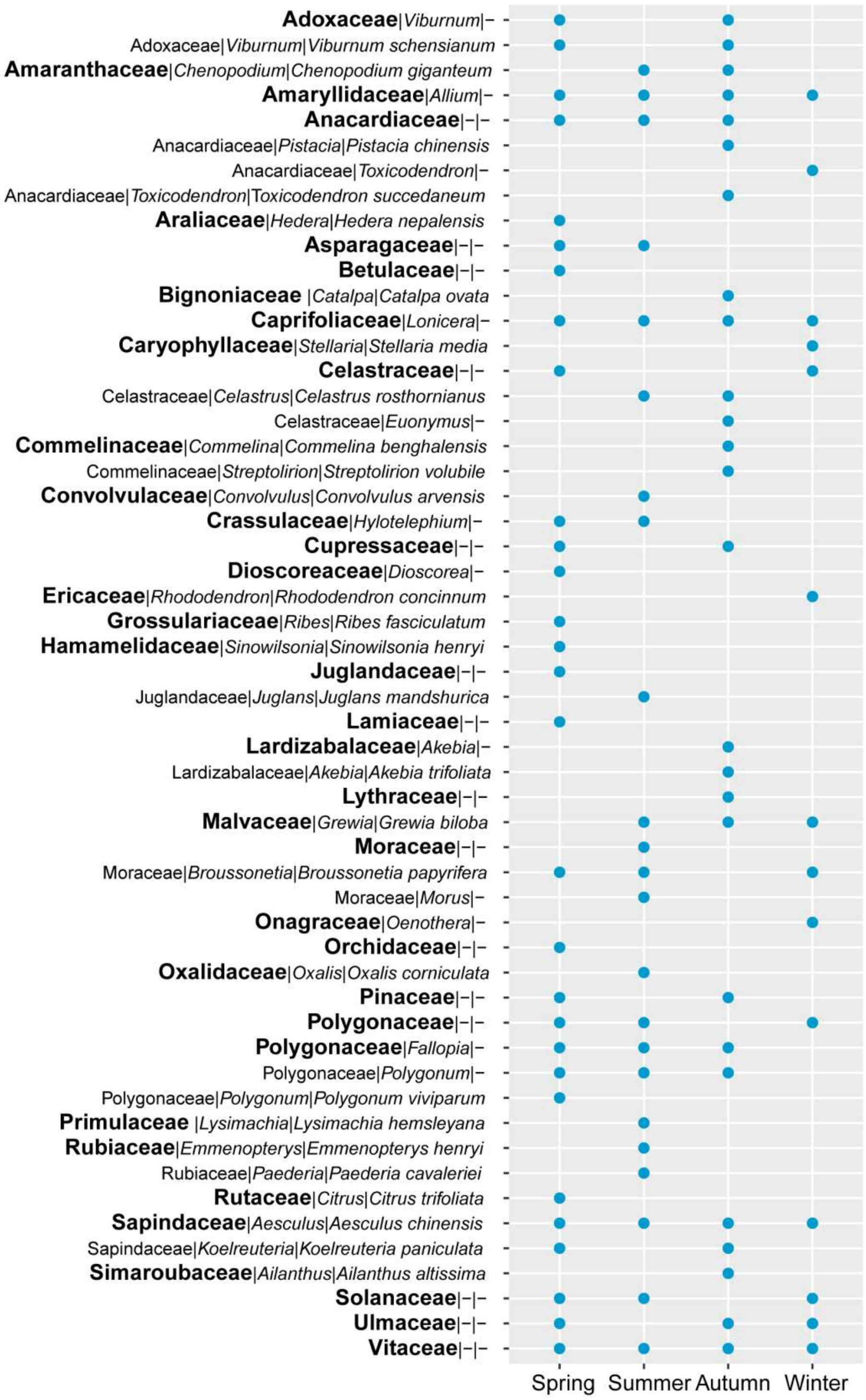

Figure S2 The taxonomic identification of food items at family/genus/species level from fecal samples of Taihangshan macaque in four seasons. "-" was represented absent taxa information.
